# Analysis of the social-epistemological dimensions of a Clinical Teaching Unit and Small Private Online Course compared to a traditional clerkship Internal Medicine

**DOI:** 10.1371/journal.pone.0316858

**Published:** 2025-01-14

**Authors:** Esther C. Hamoen, Floris M. van Blankenstein, Peter G. M. de Jong, Marlies E. J. Reinders

**Affiliations:** 1 Department of Internal Medicine, Leiden University Medical Center, Leiden, The Netherlands; 2 Center for Innovation in Medical Education, Leiden University Medical Center, Leiden, The Netherlands; Ankara University Faculty of Medicine: Ankara Universitesi Tip Fakultesi, TÜRKIYE

## Abstract

Clinical workplace learning is often suboptimal due to the dynamics of the clinical learning environment and several challenges encountered in clinical practice. At LUMC, clinical teachers introduced a novel blended learning program that included both the introduction of a Clinical Teaching Unit (CTU) and Small Private Online Course (SPOC). This study aimed to analyze the educational content and design of our educational interventions, by categorizing and comparing the dimensions of the learning program before and after the interventions. The novel blended learning program was integrated into a clerkship internal medicine, aiming to optimize clerks’ clinical workplace learning. Therefore, a traditional inpatient ward was transformed into a CTU, featuring the introduction of learning activities that promoted multidisciplinary and interprofessional learning. It included an integrated SPOC, aiming to improve clinical reasoning skills, feedback and collaboration of the clerks. The authors analyzed the clerkships’ content and design, by categorizing the social-epistemological dimensions of the teaching modes offered before (traditional clerkship) and after (including CTU and SPOC) the intervention. These dimensions consisted of individual versus group (collaborative), and objectivist versus constructive learning categories. The CTU model added eleven group activities to the clinical workplace, of which nine were characterized as constructivist-group activities. It also led to more active learning of the clerks, compared to the traditional clerkship. Analysis of the SPOC revealed 344 teaching modes in total, which included 113 objectivist–individual, 205 constructive–individual and 23 constructive–group activities. Compared to a traditional clerkship, the CTU with SPOC led to more constructivist and collaborative learning and a more diverse educational program. This study illustrates a methodology to address active and collaborative learning activities in an educational program, and offers tools to evaluate and redesign one’s teaching program. The methods and models described can be applied to clinical environments in other areas of medicine.

## Introduction

Workplace learning (WPL) is defined as learning taking place at work, through work, and for work [1]. Clinical WPL takes place through participation in actual patient care [2, 3]. It is a complex process, that is vulnerable to the dynamics and challenges within the clinical learning environment and is dependent on collaboration with other healthcare professionals [4–13].

Students encounter the clinical workplace during their clerkships. At our center, the traditional clerkship Internal Medicine is the first clerkship in the three-year Master curriculum, during which the clerks follow a 12-week clinical program in different hospitals in the area of Leiden. Each clerk has an individual program and the schedules can be quite different between distinct clerks. In general, they are appointed to different clinical departments, where they spend two to four weeks on the ward, before proceeding to another department (e.g. the in- and outpatient departments of General Internal Medicine, Endocrinology, Nephrology, Infectious Diseases, Oncology, Hematology, Cardiology, Gastrointestinal diseases, Pulmonary Diseases and Rheumatology). They attend the morning and evening reports on a daily basis, which are passive large group learning activities. They also attend formal clerkship learning activities such as (mini)-lectures. Regular learning activities for students on the patient ward mainly comprise informal activities that are related to doing ward rounds, patient admissions and consultation with supervisors or colleagues from other disciplines.

Studies show that the dynamics of the clinical learning environment not only impairs education, but also the quality of life of medical interns and patient care [14, 15]. Therefore, efforts to improve clinical WPL are critical not only for students’ learning and the quality of care they will deliver after graduation, but also for their well-being [4]. Research in this field focusses on multiple aspects of the learning environment, such as–but not limited to—student engagement, faculty development, but also interprofessional learning, and strategies for remote and online learning [16–19]. One way to promote interprofessional learning is by means of a Clinical Teaching Unit, which is a designated site for clinical education and research, with dedicated teaching staff [20]. Frequently, patient care on a CTU is provided by teams consisting of professionals with different levels of training and responsibility. Studies show a positive impact of interprofessional educational interventions on such a ward [16]. Complementary to the on-floor learning activities in a CTU, online learning can be supportive to improve the learning process for medical interns. Online learning permits flexible education at a time, place and pace convenient for the learner [21]. It can also help learners to share knowledge and experiences with others through online discussion forums and collaborative assignments, even when they are geographically dispersed. When thoughtfully designed, online learning blended with clinical learning activities, can improve education [22].

Starting in 2017, the clerkship Internal Medicine at the Leiden University Medical Center (LUMC) was adapted by two interventions that aimed to improve clerks’ clinical WPL. The first intervention was the transformation of a traditional Internal Medicine patient ward into a Clinical Teaching Unit (CTU), including the introduction of learning activities to promote knowledge, skills and multidisciplinary and interprofessional learning [23]. The transformation of our ward was accomplished by integration of educational activities into daily patient care. For this purpose, improvement of learning conditions took place by allocation of staff, time and place for teaching. Three dedicated clinical teachers, who are internal medicine specialists with an educational profile and dedicated time for clinical training, were assigned and trained. They allocated a teaching location, and enabled structured teaching time into the healthcare professionals’ work schedules. The clinical teachers and nurses’ team leaders introduced five formal learning activities that aim to stimulate multidisciplinary and interprofessional learning, and structurally scheduled them in the work week.

The second intervention was introducing blended learning to the clerkship, by integration of a Small Private Online Course (SPOC) that aimed to improve clinical reasoning skills, feedback and collaboration of the clerks [24]. A SPOC is an online course mostly used locally with a limited number of students enrolled [25].

These interventions fundamentally changed the nature of the learning environment for our clerks [23, 24]. In a traditional clerkship, learning takes place with a mix of informal and formal learning activities at the in- and outpatient clinics. Informal learning activities (implicit, unplanned and self-directed) are directly or indirectly patient-related, and includes ward rounds, the (morning) reports, several patient case discussions or meetings [26]. Formal learning activities (which are goal oriented and structured), such as mini-lectures, are also organized in the clerkship. The CTU and SPOC provided additional learning activities, mostly formal, both in the clinical and online environment, for example structural teaching visits and online patient cases respectively.

Although the CTU and SPOC concepts are not new in clinical teaching, there are no studies describing a usable methodology to analyze the educational content and design of such interventions in this context. To evaluate this post-hoc, we adopted a model by Arbaugh and Finch to analyze and categorize the contents of our teaching program [27]. The model classifies teaching modes as “instruction” (e.g. instruction video), “interaction” (e.g. discussion board), “assessment” (e.g. multiple choice questions) or “multifunctional” (e.g. virtual patient simulations) [28, 29]. Learning within these teaching modes can be further categorized in social-epistemological dimensions: two “*social dimensions”* (individual versus group) and two *“epistemological dimensions”* (transmission versus construction of knowledge). The passive transfer of information, is called an objectivistic mode, whereas knowledge construction, information processing, hands-on interaction with the content, and problem-solving, are called a constructivist teaching mode [27, 30, 31]. A teaching mode thus can be either individual or group oriented and either objectivist or constructivist.

It can be argued that group and constructivist teaching modes are indicators of good quality. Several models exist that support the hypothesis that constructive activities are better than objectivist, collaborative better than individual, and active better than passive [32]. Objectivist learning is aimed at the transfer of knowledge, while constructive learning is defined as learning that is meaningful, in which the learner actively builds a mental model of the system to be learned, which facilitates learners build new knowledge upon the foundation of previous learning [33–35]. It simulates deeper understanding and embedding of information, through a more active acquisition of knowledge [31, 35, 36]. Interaction between learners during collaboration enables them to build knowledge upon another’s understanding [34]. Group-oriented, cooperative learning activities with peer interaction can therefore lead to a restructuring of thinking and the construction of new knowledge [37]. In active learning, learners activate their own knowledge within the boundaries of a certain content by doing overt learning activities, instead of passively receiving information [32, 34].

While previous research on this topic mainly investigated online courses [28–30], the social-epistemological dimensions approach used by Arbaugh and Finch might also be used to classify and analyze the quality of the face-to-face learning activities in the clinical workplace. The aim of this study was to use the Arbaugh and Finch model to evaluate the contents and design of teaching modes before and after introducing a CTU and SPOC.

## Methods

### Context

In an attempt to improve the quality of the clinical WPL in this clerkship, the traditional Internal Medicine inpatient ward was transformed into a CTU. As a result, the clinical teachers and nurses’ team leaders introduced five formal and structurally planned learning activities on this ward, that were in addition to the other traditional clerkship activities: a teaching visit, pre-rounds, skills training, clinical lessons and a multidisciplinary grand round [23]. These activities aimed to specifically stimulate multidisciplinary and interprofessional learning. A teaching visit is a training session that involves four to six clerks and a clinical teacher. One of the clerks performs the history taking and physical examination with an unfamiliar patient, and is observed and assessed by the peers and teacher. During pre-rounds, three clerks autonomously present a patient report, execute the ward rounds, and formulate a treatment or diagnostic plan in the patient file, a process that is observed and assessed by peers and a clinical teacher. Skills training involves a nurse-educator who instructs the clerks about skills performed in medical practice (like insertion of intravenous catheters or feeding tubes) and subsequently observes and assesses the specific skill executed. A Clinical lesson is a didactic session presented by a clinical teacher or other physician, or nurse. A multidisciplinary Grand Round is a weekly session attended by clerks, residents, supervising clinicians, and residency program directors of different disciplines involved in the ward. Complex patient cases requiring involvement of different disciplines or interesting patient cases for educational purposes can be presented and discussed.

Additionally, the teachers introduced a Small Private Online Course (SPOC) into the clerkship. The course was developed using a design-based research approach in which practical and theoretical aspects were integrated in the educational design. During a learning experience design session, a group of identified stakeholders set the course’s goals, students’ needs, learning goals and aimed “look and feel.” They used the outcomes of this session to define the exact content and learning activities centered around authentic clinical problems that are typically encountered in internal medicine. Learning activities contained aspects of constructive, collaborative and contextual learning and aimed at stimulating students’ self-regulated learning and motivation during the course [24]. The SPOC consisted of three parts: “getting ready for the clerkships”, “getting ready for internal medicine” and “into patient care.” Clerks were supposed to do weekly assignments asynchronously, with compulsory and optional features to meet their varying needs during their clerkship. The SPOC included virtual patient cases, group-assignments, discussion forums for peer-feedback sessions, quizzes, augmented and virtual reality assignments, and background information. In our clerkship, the SPOC was used to blend online learning with clinical WPL, with the ultimate goal to improve clinical reasoning skills, feedback and collaboration of the clerks. We evaluated the SPOC and student perceptions in a pilot study after implementation [24].

### Study procedure

In this study, we used a social-epistemological dimensions model to compare teaching in a CTU with integrated SPOC with teaching in a traditional patient ward [27–30]. Consequently, the study is a document analysis, and does not involve any human subjects or human tissue. Ethics approval and the need for informed consent were waived by the Educational Research Review Board of LUMC.

From December 2021 –May 2022, a data collection file was composed to record the number of instructional, interactional, active processing and multifunctional modes present in the CTU model and SPOC, compared to a traditional inpatient ward. This table was based on previous studies, where the mode ‘assessment’ was replaced by ‘active processing’ to fit the CTU activities and SPOC assignments more realistically [28–30]. We made distinction between active and passive learning, since observation of a learning activity is distinct from active participation in a learning activity.

All distinct learning activities at the traditional ward and CTU were determined and categorized in teaching mode profiles. For the SPOC, the first three investigators, in their roles as clinical teacher (EH) and educational experts (FvB and PdJ), logged into the SPOC learning environment to score the online assignments. All three, individually assed all teaching modes. Each investigator categorized the contents of one learning activity as a certain type of teaching mode. They compared their outcomes followed by a joint review of the activity and discussion if necessary, until they obtained full consensus. All steps were tracked in a data log. After scoring the first eight lessons (in the first and second month of the clerkship), the three investigators reached immediate agreement on the categorization, whereafter the first two investigators (EH and FvB) finalized the scoring of the remaining assignments. The first two authors allocated the social-epistemological dimensions, and the outcomes were thereafter discussed with the third investigator (PdJ) until consensus was achieved. Most learning activities could be classified in the predefined tables, however some new teaching modes had to be added to fit some specific learning activities in the SPOC.

## Results

### Teaching modes and social-epistemological dimensions of the CTU

We identified thirteen teaching modes in the traditional patient ward, and twenty-five distinct teaching modes in the CTU. Table 1 presents the different teaching modes in the respective wards. The learning activities for clerks in a traditional patient ward mainly comprised informal, passive learning by observation of other healthcare professionals executing their tasks related to direct patient care. Clerks played an active role during the rounds (such as taking a patients’ history, physical examination, reporting the patient file), however the more communicative activities such as the morning report, consultation with the supervisor and communication with the patients’ family was mainly performed by the residents and rarely by the clerks.

**Table 1:** Teaching modes and social-epistemological dimensions of the CTU compared to a traditional patient ward.

Teaching mode profile	Social-epistemological dimension		Comments
	Traditional Ward	CTU	
**Instruction**			
1. Morning report	OG	OG	
2. Evening report	OG	OG	
3. (Mini)-lectures	OG	OG	
*4*. *Clinical lesson*	x	OG	
*5*. *Instruction skills*	x	OG	
**Interaction**			** **
6. Ward round (interaction nurses, resident, supervisor and patient)	OG	CG	Shift passive > active
7. Grand round (interaction with supervisors)	OG	CG	Shift passive > active
8. Patient admission (interaction patient)	CI	CI	
9. Family consultation	OI	OI	
10. Multidisciplinary consultation	OG	OG	
*11*. *Preround (interaction with patient)*	*x*	CG	
*12*. *Teaching visit (interaction with patient)*	*x*	CG	
*13*. *Multidisciplinary grand round (interaction with other subspecialists)*	*x*	OG	
**Active processing**			** **
14. Ward round (training history taking and physical examination skills)	CG	CG	
15. Ward round administration and make up a patient file	CI	CI	
16. Grand round (reporting a clinical case and suggesting a treatment plan)	OG	CG	Shift passive > active
17. Patient admission (training history taking and physical examination skills)	CI	CI	
*18*. *Preround (training history taking and physical examination skills)*	*x*	CG	
*19*. *Teaching visit (training history taking and physical examination skills)*	*x*	CG	
*20*. *Multidisciplinary grand round (reporting a clincal case and suggesting a treatment plan)*	*x*	CG	
*21*. *Execution of skills (individual)*	*x*	CI	
*22*. *Execution of skills (group)*	x	CG	
**Multifunctional**			** **
23. Observation resident during informal learning activities*	OI	OI	
*24*. *Pre-round (observation peers)*	x	OG	
*25*. *Teaching visit (observation peers)*	x	OG	

X = not present, OI = objectivist—individual, OG = objectivist—group, CI = constructivist—individual, CG = constructivist—group. * ward round, MDC, family consultation. *In italics*: additional learning activities in CTU model.

We identified additional passive and active learning activities in the CTU. These included different activities during ward rounds, the weekly grand round, pre-round, teaching visit, clinical lessons and skills and all involved direct patient contact.

Allocation of the social-epistemological dimensions to the above described teaching modes, revealed that learning in a traditional ward was mainly accomplished by objectivist-group (OG) activities. The CTU model added several group learning activities to the program, that were mostly constructive (CG). It also led to a shift from a passive to active involvement of the clerks in some learning activities, as observed in our previous study [23].

### Teaching modes and social-epistemological dimensions of the SPOC

Table 2 represents an overview of the teaching modes addressed in the SPOC, that were categorized in social-epistemological dimensions. The SPOC includes a total of 344 teaching activities, that comprised eighteen distinct types of teaching modes; seven instructional modes, three interactional modes, seven active processing modes and one multifunctional mode.

**Table 2:** Teaching modes and social-epistemological dimensions within the SPOC.

Teaching mode profile	Getting Ready For the Clerkships	Getting Ready For Internal Medicine	Into Patient Care	TOTAL COURSE	Social-epistemological dimension
**Instruction**	**n**	**n**	**n**	**n**	** **
1. Digital text or textbook	16	8	18	42	OI
2. Illustrations or simulations	2	1	26	29	OI
3. Links to external online resources	12	13	116	141	CI
4. Prompts to use external link for activities in the course		1	9	10	CI
5. Virtual reality 360° video	2		1	3	CG
6. Anamnesis video			7	7	OI
7. External embedded video	5	1	18	24	OI
**Interaction**					
8. Discussion board posts answering questions prompted	2	1	14	17	CI
9. Prompts to respond to peers			12	12	CG
10. Group assignment			2	2	CG
**Active processing**					
11. Multiple Choice Question		5	6	11	OI
12. Ranking question			2	2	CI
13. E-learning		3	3	6	CI
14. E-tivity			2	2	CI
15. Peer reviewed open ended question with long answer			6	6	CG
16. Open ended question with long answer	3	1	22	26	CI
17. Survey			1	1	CI
**Multifunctional**					
18. Virtual patient case			3	3	CI
**Grand total**				**344**	

OI = objectivist—individual, OG = objectivist—group, CI = constructivist—individual, CG = constructivist—group

These teaching modes were categorized in social-epistemological dimensions, indicating that the SPOC has added five objectivist–individual (OI), no OG, nine constructivist–individual (CI) and four CG modes to the clerks’ learning program.

**Fig 1:**
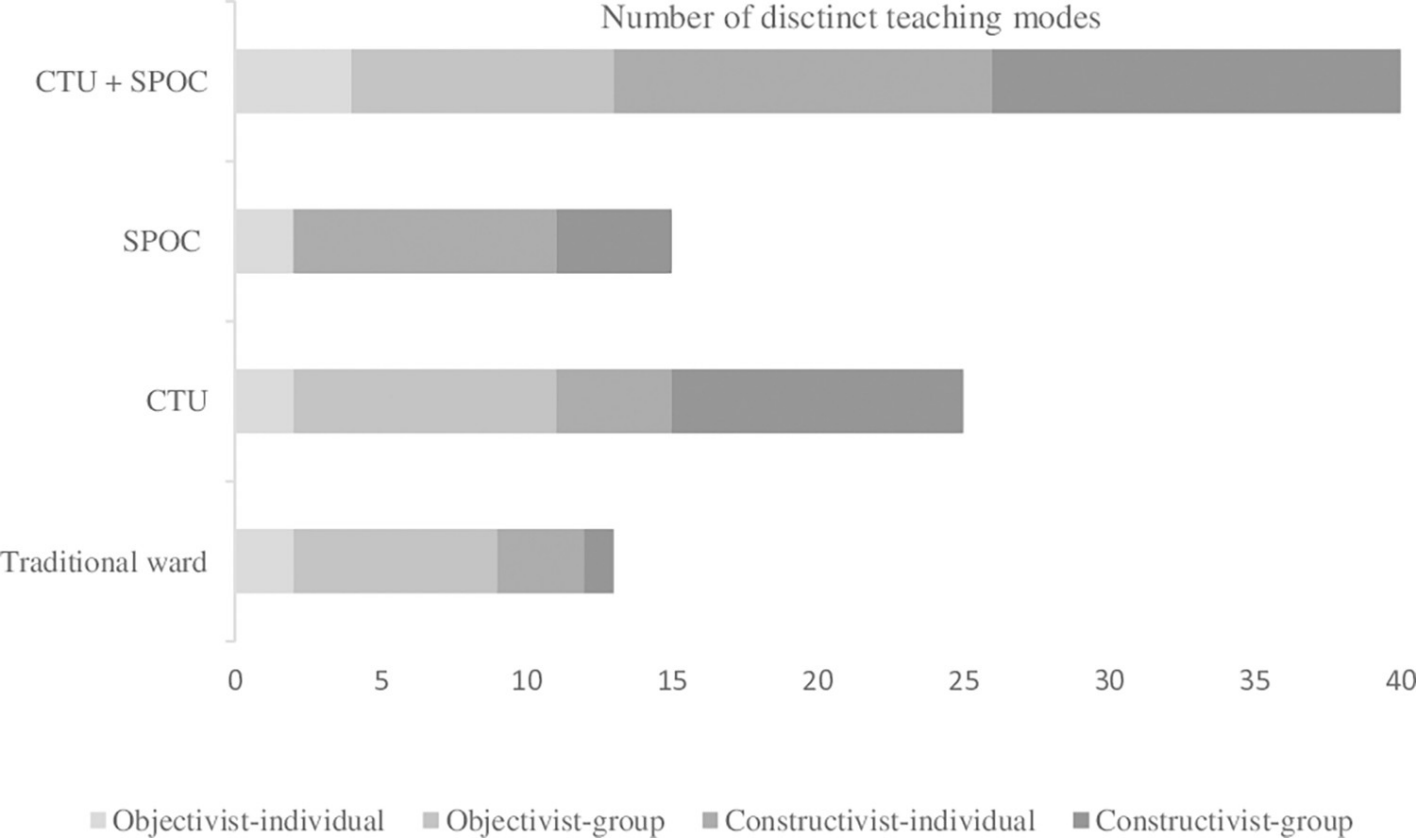
An overview of the number of teaching modes in different learning environments. Displayed are the distinct teaching modes in a traditional ward, CTU, SPOC and the CTU/SPOC combined.

An overview of the number of distinct teaching modes in a traditional ward, a CTU, SPOC and the latter two combined (which presents the new learning environment after the interventions) is displayed in Fig 1.

## Discussion

This study aimed to evaluate the instructional quality of the clerkship Internal Medicine by analyzing and characterizing the teaching modes and social-epistemological dimensions of the traditional clerkship Internal Medicine (before) and after integration of a CTU and SPOC (intervention). Up to our knowledge, this is the first study using this model in this clinical and online context. The intervention added several new teaching modes to the educational program of our clerks internal medicine, through introduction of online learning activities in the SPOC and formal learning activities in the CTU model. Compared to a traditional ward, the SPOC and CTU added eighteen and twelve distinct teaching modes respectively, and three modes have changed from OG to CG activity by changing the role of the clerk from observer to active learner. This has enriched the clinical workplace learning in our clerkship.

The social-epistemological evaluation showed that the traditional clerkship mostly contained objectivist–group activities and a limited constructivist learning activities. The CTU model had several forms of group learning, which have predominantly a constructivist approach. In addition, integration of the SPOC in the existing curriculum added several constructivist online learning activities to the educational program, and also some group activities.

Arbaugh and Finch conclude that collaboration should be the foundation of the design and delivery of online courses. Other studies using this model for evaluation of online courses specifically, confirm the need for collaborative learning strategies [28, 29, 38]. In the current study, the model is used to evaluate both the clinical learning activities in the CTU as well as the online activities in the SPOC [27]. Other literature supports the hypothesis that constructivist learning is better than objectivist, collaborative better than individual, and active better than passive [27, 32, 39–43]. Therefore, we argue that from this viewpoint the educational quality of our educational program has been improved by the interventions, although student evaluation would be necessary to investigate this.

One needs to realize that the presence of constructive and collaborative learning modes alone may not be sufficient to improve learning; other learning conditions should also be taken into account, particularly in online learning environments. Research suggests that favorable conditions for online learning are a good balance between course structure and student autonomy, and active monitoring and guiding of students by teacher(s) [40]. Other research shows that the quality of communication can decrease in virtual discussions in online courses [44, 45], which may decrease the positive interdependence (students providing actions to promote the achievement of shared goals) that is a key for successful collaborative learning [45–47]. Failure to implement the required conditions in online courses and suboptimal communication within the course may affect the learning process, even if elements of constructive and collaborative learning are present. Whether the integration of our new program has led to improved collaboration, could be subject to further research.

Beside constructive and collaborative learning activities, the new program has also added objectivist and individual activities, which potentially add specific benefits to the program. Embedding different social-epistemological dimensions in a course can be helpful for teaching specific contents; while for complex content, group learning is more suitable than individual learning, however for simpler content individual learning can be more effective [48]. Objectivist modes can be preferred over constructivist modes when time is limited because the latter require more advanced knowledge, comprehension and teacher feedback [49]. Thus, the right mixture of teaching modes in a course can be helpful to fulfill different needs of the learner and support teaching of the specific campus contexts and aims of the course, and can even can be supportive for successful integration of the course in the existing curriculum [28].

### Limitations

This study has some limitations. Informal learning activities on a traditional patient ward may vary between different wards, hospitals and may even be totally different compared to other countries. In other words, the study took place in one specific context and it is not known if our results can be transferred to another context. We also did not assess if the required conditions for effective online learning were met.

In this study, the learning outcomes, perceptions of the intervention and student satisfaction have not been addressed, which could be subject to future research. Further research using social-epistemological dimensions in the development, integration and analysis of blended curricula is needed to obtain more insight in the implications, strengths and weaknesses of using such a methodology.

## Conclusions

From our study it can be concluded that the implementation of the CTU and SPOC in our clinical clerkship Internal Medicine increased the number of distinct teaching modes, particularly of the constructivist sub-types. In addition, the current study shows that the methodology used may not only be suitable for analyzing online learning environments, but also the learning activities offered in clinical WPL. For teachers implementing new learning activities, this study has several implications. Analysis of one’s curriculum or learning activities within a social-epistemological dimensions model helps to objectively evaluate the program that is offered. This methodology can also be used as a theoretical base while developing new learning activities. It is a tool to integrate active, collaborative and constructive learning in an existing educational program, and facilitate the program to meet specific individual or contextual educational needs, to support resources for education and to optimally integrate in an existent curriculum. Our data may also support medical educators and faculty in gathering resources to focus on student centered, active and collaborative learning strategies to improve their curriculum. The study is of value by setting an example for using a solid methodology to address active and collaborative learning activities in an educational program.

## Supporting information

S1 Raw data (TIF)

## List of abbreviations

CG: Constructivist–group
CI: Constructivist—individual
CTU: Clinical Teaching Unit
LUMC: Leiden University Medical Center
OG: Objectivist–group
OI: Objectivist—individual
SPOC: Small Private Online Course
WPL: Workplace learning
