# Supplementary figures and images for: Analysis of the social-epistemological dimensions of a Clinical Teaching Unit and Small Private Online Course compared to a traditional clerkship Internal Medicine
Source: PLoS One. 2025 Jan 14;20(1):e0316858. doi: 10.1371/journal.pone.0316858 (PMC11731731; doi:10.1371/journal.pone.0316858)

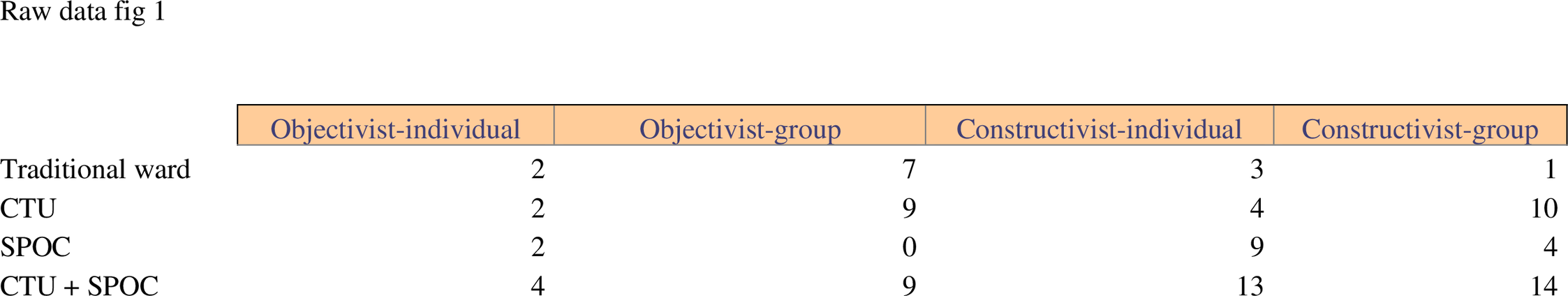

Supplement: S1 Raw data — (TIF) [file pone.0316858.s001.tif]
